# Voice-Based Early Diagnosis of Parkinson’s Disease Using Spectrogram Features and AI Models

**DOI:** 10.3390/bioengineering12101052

**Published:** 2025-09-29

**Authors:** Danish Quamar, V. D. Ambeth Kumar, Muhammad Rizwan, Ovidiu Bagdasar, Manuella Kadar

**Affiliations:** 1 Department of Computer Engineering, Mizoram University, Mizoram 796004, India; danishquamarcse@gmail.com; 2 Data Science Research Centre, College of Science & Engineering, University of Derby, Derby DE22 1GB, UK; m.rizwan@derby.ac.uk; 3 Department of Mathematics, Faculty of Exact Sciences, “1 Decembrie 1918” University of Alba Iulia, 510009 Alba Iulia, Romania; 4 Centre of Research Project Management “1 Decembrie 1918” University of Alba Iulia, 510009 Alba Iulia, Romania; mkadar@uab.ro

**Keywords:** Parkinson’s disease, speech processing, ML, BiLSTM, DL, CNN

## Abstract

Parkinson’s disease (PD) is a progressive neurodegenerative disorder that significantly affects motor functions, including speech production. Voice analysis offers a less invasive, faster and more cost-effective approach for diagnosing and monitoring PD over time. This research introduces an automated system to distinguish between PD and non-PD individuals based on speech signals using state-of-the-art signal processing and machine learning (ML) methods. A publicly available voice dataset (Dataset 1, 81 samples) containing speech recordings from PD patients and non-PD individuals was used for model training and evaluation. Additionally, a small supplementary dataset (Dataset 2, 15 samples) was created although excluded from experiment, to illustrate potential future extensions of this work. Features such as Mel-frequency cepstral coefficients (MFCCs), spectrograms, Mel spectrograms and waveform representations were extracted to capture key vocal impairments related to PD, including diminished vocal range, weak harmonics, elevated spectral entropy and impaired formant structures. These extracted features were used to train and evaluate several ML models, including support vector machine (SVM), XGBoost and logistic regression, as well as deep learning (DL)architectures such as deep neural networks (DNN), convolutional neural networks (CNN) combined with long short-term memory (LSTM), CNN + gated recurrent unit (GRU) and bidirectional LSTM (BiLSTM). Experimental results show that DL models, particularly BiLSTM, outperform traditional ML models, achieving 97% accuracy and an AUC of 0.95. The comprehensive feature extraction from both datasets enabled robust classification of PD and non-PD speech signals. These findings highlight the potential of integrating acoustic features with DL methods for early diagnosis and monitoring of Parkinson’s Disease.

## 1. Introduction

Parkinson’s disease (PD) is a progressive neurodegenerative disorder mainly affecting motor functions. The disorder was first extensively described in 1817 by the British physician James Parkinson in his book “An Essay on the Shaking Palsy” [1]. Parkinson described the key symptoms such as resting tremor, bradykinesia (slowness of movement), rigidity and postural instability. Though his description was groundbreaking, the disease was poorly understood for decades. The French neurologist Jean-Martin Charcot broadened clinical knowledge of the disease in the late 19th and early 20th centuries, distinguishing it from other types of motor disorders [2]. In modern times, PD research has aimed to understand its complex etiology and create disease-modifying treatments. As most of the PD cases are classified as idiopathic, pathogenic genetic factors have been recognized in 10–15% of PD cases. Mutations of genes like LRRK2, PINK1, PARK7 and SNCA are associated with familial Parkinson’s. Today’s research examines the interaction between genetics and environment, where factors like pesticide exposure or heavy metals come into play. Recent developments in self-supervised learning (SSL) have introduced powerful speech models such as Wav2Vec 2.0 and HuBERT (Hidden unit BERT) [3]. These models learn general representations of speech from large-scale unlabeled data and have demonstrated state-of-the-art performance in tasks such as speech recognition, speaker identification, and clinical voice analysis. Unlike traditional handcrafted features such as MFCCs, jitter, and shimmer, SSL models capture both acoustic and contextual information directly from raw waveforms, reducing the need for manual feature engineering. Their successful application in health-related domains suggests strong potential for detecting PD and related speech impairments, motivating their inclusion in future research directions. Voice and speech impairment is among the earliest non-motor symptoms of PD, often manifesting as hypokinetic dysarthria, a cluster of speech abnormalities including reduced vocal loudness, monotonic pitch, breathiness, imprecise articulation, and irregular speech rate [4]. Hypokinetic dysarthria is a cluster of speech features typical of the disorder including a decrease in vocal loudness, difficulty modulating pitch (monotonous), breathy voice quality, poorly articulated sounds (dysarthria) and irregular speech rate. For this reason, voice analysis has emerged as a valuable, innocuous tool for the early diagnosis and follow-up of PD.

In recent years, several new trends in the use of artificial intelligence (AI), ML and DL techniques are reported for Parkinson’s recognition through observed changes of speech patterns and vocal features [5]. Acoustic features like jitter, shimmer, harmonics-to-noise ratio (HNR), MFCCs, formant frequencies and vowel space area can be extracted alongside the speech recordings by researchers. These features are then used to feed ML/DL models such as SVM, random forests, CNNs and RNNs to classify PD subjects against non-PDs.

A new wave of technologies including mobile health apps and wearable gadgets are beginning to integrate voice analysis tools [6]. These systems allow for constant, real-time monitoring of patients in the real-world environment, enhancing accessibility while reducing the need for hospital visits.

Explainable AI (XAI) is another trend in PD voice detection that is on the upswing. Techniques such as SHAP (SHapley Additive exPlanations) and LIME (Local Interpretable Model-Agnostic Explanations) aid in understanding the contribution of various vocal features to the classification decision, enhancing the transparency and reliability of these models [7]. PD is a coin with a long history from the name itself, through the first clinical descriptions, to cutting-edge research today. Among them, voice analysis appears as a useful and effective simple technique for Parkinson’s disorder screening and monitoring. Innovations such as AI, DL, mobile technology and explainable models are paving the way for a better future for PD patients [8]. Providing a better way to understand it, a better way to measure it, a better way to overcome the obstacles to diagnosis and a better way to live with hope.

ML is the branch of artificial intelligence that allows computers to learn from specific training data and create analysis models without relying on predetermined formulas. Unlike traditional programming, ML algorithms identify patterns in data and improve their accuracy as more data becomes available. The main types of ML include monitored learning, which uses classified data for classification and return tasks; noteworthy learning, which discovers hidden patterns in unclassified data through techniques such as clusters; and strengthening learning, which helps to understand decisions with experiences and interactions with an environment.

ML has many applications, including healthcare, financial services and cyber security. With the right lessons on amazing amounts of data, machine learning models can find problems and solve them quickly. Deep learning is another way of machine training. This is also the way of teaching advanced methods, but it focuses on using artificial neural networks. These networks are similar to neurons in the human head.

Despite these advances, challenges remain in generalization across datasets, interpretability of models, and deployment in real-world scenarios. Our work builds on these foundations by combining spectrogram-based features with recurrent deep learning architectures, aiming to provide a more comprehensive evaluation and robust performance in PD voice classification.

## 2. Organization of the Paper

The structure of this paper is outlined as follows:

### 2.1. Introduction

Background of Parkinson’s disease and the motivation for using voice analysis.

### 2.2. Related Work

Summarizes previous studies in a comparative table including datasets, models, performance metrics, limitations and contributions.

### 2.3. Research Design

This study relies on secondary data obtained from datasets available on Figshare. The data was pre-processed to remove noise and normalize the recordings. Feature Extraction: Speech samples were converted into spectrograms and analyzed for features such as MFCC, jitter, shimmer and RPDE. Classification: The audio/visual samples were classified using CNN+LSTM, CNN+GRU, BiLSTM, deep neural networks (DNNs) and traditional ML methods.

### 2.4. Results and Discussion

Presents the experimental findings: BiLSTM achieved 97% accuracy, CNN + GRU 94%, CNN + LSTM 91% and DNN 81%. Traditional ML methods (SVM, XGBoost, logistic regression) achieved approximately 44–55% accuracy. Figures and tables comparing deep learning and machine learning approaches are provided.

### 2.5. Conclusions

Summarizes the contributions of this study, emphasizing the effectiveness of spectrogram-based features combined with deep learning models for early PD detection and notes directions for future research.

## 3. Related Work

Costantini et al. [9] compared voice assessments for patients with Parkinson’s disease both off and on L-Dopa treatments. This revealed that the traditional ML models showed ascertained the expectations, even though the CNNs were used in the state-of-the-art systems, especially in the binary classification. The study used vocal recordings to identify Parkinson’s disease and found that features like pitch and prosody-based elements could be effective biomarkers. The CFS method was used to select the most relevant features, improving system accuracy. Overall, the study indicated that ML models such as k-nearest neighbors (KNN) and SVM with greater-specific data work better than deep learning methods.

Yadav et al. [10] suggested an AI-based model for the diagnosis of Parkinson’s disease using feature selection strategies such as Chi-square (χ^2^), extra trees and correlation matrix, combined with many supervised classifiers. Their study showed that decision trees achieved maximum accuracy (94.87%) with strong ROC performance (AUC = 98.7%), emphasizing the opportunity to approach based on ML for a reliable and quantitative PD diagnosis.

Ali et al. [11] highlighted that speech analysis provides non-invasive and promising methods for diagnosing Parkinson’s disease, where acoustic properties such as tone, jitter, shimmer and formant are used for ML and DL classification. Their review focuses on supporting neurologists, especially showing strength in identifying PD with timely and accurate help. Nijhawan et al. [12] proposed a new transformer-based approach for the detection of Parkinson’s disease using dysphonia measurements from voice recordings. Unlike typical models based on providing decisions, their neural network framework supports continuous learning and multimodal integration, which advances state-of-the-art performance. The model achieved superior results compared to gradient-boosted decision trees (GBDTs), with at least 1% improvement in AUC and increased accuracy and recall. In addition, they introduced an XGBoost-based feature selection strategy and showed the strength of the transformer over simple MLP in learning complex vocal features.

Pradeep Reddy et al. [13] conducted a comprehensive study on Parkinson’s disease with AI-based diagnosis using voice measurements from the Oxford PD dataset. They evaluated 26 machine learning algorithms, including logistic regression, mapping trees, SVM, random forest, boosting techniques and neural networks. Among them, multi-layer perceptron (MLP) achieved the best performance with 95% accuracy, 94% precision, 100% sensitivity, F1 score 97% and AUC 98%. Their findings emphasize the strong ability of MLP to distinguish between PD patients from healthy people and show the importance of AI in clinical decisions and early interventions. Hoq et al. [14] proposed two hybrid models to classify vocal features for the detection of Parkinson’s disease. The models included PCA-SVM and SAE-SVM. Among the two, the model with superior performance was the one that used sparse autoencoder (SAE) for feature compression followed by SVM classification. The hybrid SAE-SVM model achieved the highest accuracy of 93.5%. Along with that, it provided an F1-score of 0.951 and MCC of 0.788. The model also outperformed other standard classifiers such as MLP, XGBoost, KNN and RF. The study effectively addressed data imbalance using SMOTE in vocal datasets. This study concluded that the hybrid SAE-SVM model provides a highly effective and reliable solution for the early diagnosis of PD based on vocal impairments.

Karaman et al. developed [15] deep CNN models for automatic detection of Parkinson’s disease using voice signals developed from the mPower database. They obtained DenseNet161 as the most effective model by applying fine adjustment with transfer growth on SqueezeNet 1_1, ResNet101 and DenseNet161. The accuracy achieved was 89.75%, sensitivity was 91.50% and precision was 88.40%. This research suggests that CNN-based techniques can exceed traditional methods for Parkinson’s diagnosis. This approach also provides opportunities to integrate smart devices, which can help patients obtain clinical support quickly before diagnosis. Bukhari et al. [16] proposed an ensemble machine learning framework for the diagnosis of Parkinson’s disease using speech signals and emphasized the effectiveness of computational methods compared to traditional diagnostic methods. The model made a classifier AdaBoost and trained on the UCI PD dataset, including different vocal features such as MFFC, wavelet changes and quality of shaking waveform. The system achieved strong performance with AUC 0.99, accuracy of 0.96 and a strong flexibility recall balance. The study shows that combined ML techniques can provide reliable and constant early PD diagnosis.

Kiran Reddy et al. [17] presented and examined an exemplary based sparse representation (SR) approach for the detection of Parkinson’s disease from speech. The SR method for acute disease avoids the complex training required in conventional ML models and tunes hyperparameters, which makes it a simpler alternative. This approach also provides stability against noise and redundancy in data. The study used L1-accounting least squares (L1-LS) and non-negative least squares (NNLS) to implement a class-specific vocabulary structure designed to increase the decomposition of superficial structures. It helps improve accuracy. Results showed that the SR approach based on NNLS revealed higher performance compared to conventional ML methods, both in PC-Gita and mobile voice datasets. This study emphasizes the potential of SR approaches in creating reliable and non-invasive PD screening tools.

Kavita Bhatt et al. [18] proposed a framework for the detection of Parkinson’s disease based on deep neural networks (DNN), using spectrograms produced by superlet transformation (SLT). SLT effectively used 1-D speech signals in 2-D spectrograms, which were classified using InceptionResNetV2, VGG-16 and Resnet50v2. Their experiments with the PC-GITA and Italianpvs datasets showed excellent performance, with VGG-16 achieved 92% accuracy higher than expected on measured vowels and 96% on Italianpvs. The study emphasized that the short technique-based approach achieved more than traditional methods such as Hilbert spectrum, Emd, Cwt and Stft for PD speech detection.

Madhu Yagnavajjula et al. [19] proposed system uses the Saarbruecken voice disorder database, where the WST-based features combined with SVM and neural network classifiers achieved superior performance in both binary and multi-class classification tasks. This study showcased that WST-based features outperformed conventional approaches, providing a reliable framework for detecting neurological voice disorders. A method for automatic classification of voice disorders based on wavelet scattering transform (WST) features has been developed. System WST relies on the ability to generate waves that allow scale-based localisation of frequency changes that help in identifying dysphonic voices. WST prepares very robust representations by means of convolution, modulus and averaging in the wave forms that protect variations intra-class while increasing class separability.

Below Table 1 summarizes the techniques used, dataset details and limitations of the related works.

## 4. Material and Method

### 4.1. Dataset of Parkinson Disease

The primary audio dataset was collected from the publicly available repository on Figshare. It is a collection of the recordings of 81 individuals which includes 41 non-PDs (Hc) and 40 people with PD (PwPD). The participants’ ages vary from 18 years to 85 years with a mean age of 57.26 years. The dataset contains males and females, with females being the majority with a mean age of 44 years. The voice recordings were stored in .wav format and records participants holding the pronunciation of the vowel /a/. The phonic recordings were captured via participants’ own telephones distanced across thousands of miles from each other facilitating access and participation. At the time of the data acquisition, the audiometric data subjects were requested to extend the vocal vowel /a/ for as long as possible and with a similar tone and amplitude. The approach is common in speech analysis studies to identify vocal variations related to neurological vocalization diseases, including PD.

Second dataset consists of 15 participants (5 PD and 10 non-PD) from Mizoram, India, aged 20–60 years (10 men and 5 women). Registration was done using the mobile phone microphone in a quiet, controlled environment, with equipment located approximately 10–15 cm from the speaker’s mouth. Each participant performed a task on the words by pronouncing the words of the alphabet. The signals were stored in .wav format with a frequency of 44.1 kHz, 16-bit resolution and mono channel. The dataset summary is shown in Table 2.

This dataset is considered a pilot feasibility collection only. Due to its limited size, Dataset 2 was not included in the training or testing of models in this study. Instead, it demonstrates the practicality of mobile-based data collection. Removing Dataset 2 entirely was considered but retained only because it provides contextual and future value and will be expanded in future work.

### 4.2. Dataset Description and Preprocessing

- Dataset 1: The recordings were obtained with a sampling frequency of 8 kHz (telephone-quality). An effective anti-aliasing cutoff was applied at approximately 4 kHz, and subsequent analysis was limited to the 0–4 kHz frequency range, which is typically investigated in PD speech studies.
- Dataset 2: The recordings were sampled at 44.1 kHz with 16-bit resolution, providing higher fidelity for spectral feature extraction.
- Sequence duration: Since the recordings were of varying lengths, fixed-length segmentation/padding of 3 s was applied to ensure uniform input across all samples during model training.

All methods were carried out according to the relevant guidelines and regulations. The voice analysis experimental protocols were conducted by the ethical standards of Mizoram University and approved by the Institutional Review Board (MZU/CE/PHD IRB 005). Informed verbal consent was also obtained from all participants before their engagement in the study.

### 4.3. Attributes for Voice Detection in Parkinsons Disease

The extracted features represent [20] several voice attributes used to detect and categorize speech impairments caused by PD using audio analysis. These features can be grouped into temporal, spectral, cepstral and nonlinear properties. The summary of speech attributes are shown in Table 3.

#### 4.3.1. Time Domain (Temporal) Features

- **Jitter**: Quantized changes in pitch (irregularity in vocal folds).
- **Shimmer:** Assesses the change in amplitude, which indicates voice tremor and instability.

#### 4.3.2. Frequency-Domain Features

- **Fundamental frequency (F0)/Pitch**: Shows the speed at which the vocal cords vibrate. In patients with Parkinson’s disease, this can be unstable.
- **Harmonics-to-noise ratio (HNR)**: Evaluates the ratio of harmonic expression to noise in speech, often reduced in PD.

#### 4.3.3. Frequency-Domain (Spectral) Features

To extract features, the first one from the time domain is [21]:

- **Mel-frequency cepstral coefficients (MFCCs)**: Captures the speech spectrum and is widely used in ML models.
- **Spectral centroid**: Decline in PD spectrum causes a shift in the center of mass of the spectrum.
- **Spectral bandwidth**: The spread of the spectrum, generally lower in PD patients.
- **Spectral contrast**: Distinguishes between peaks and valleys in speech signals, helpful in detecting PD-related voice changes.

#### 4.3.4. Cepstral Features

- **Cepstral peak prominence (CPP)**: Measures the strength of the periodic component in speech.
- **Linear predictive coding (LPC)**: Assists in modeling the vocal tract and identifying articulation impairments.

#### 4.3.5. Non-Linear Features (Voice Stability, Complexity)

- **Recurrence quantification analysis (RQA)**: Quantifies complexity and stability of vocalization patterns [22].
- **Lyapunov exponents**–Measures the chaotic behavior of speech, proportional to the extent of aberrant vocal fold vibration.
- **Glottal-to-noise excitation ratio (GNE)**: Explains how noisy the glottal source signal is.

### 4.4. Proposed Methodology

**Figure 1:**
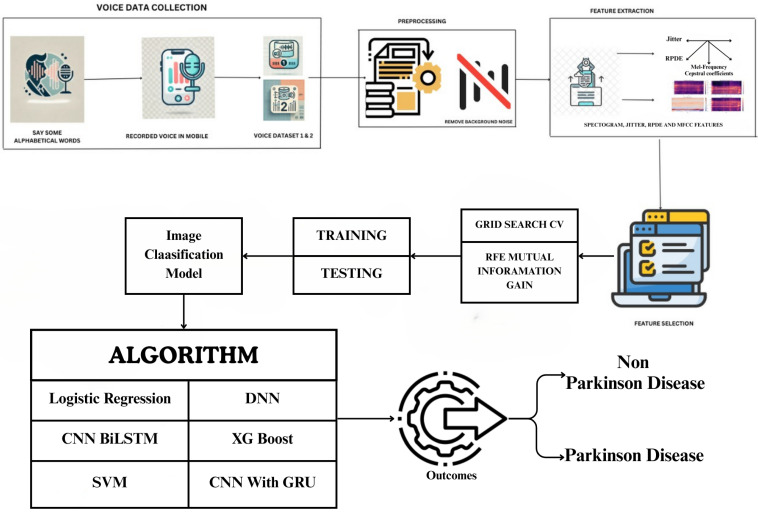
Proposed methodology.

Voice datasets for PD detection were utilized in this study. The first dataset was obtained from Figshare [23], containing voices of 81 subjects made up of 41 non-PDs (Hc) and 40 subjects with PwPD where individuals pronounced the vowel /a/. The second dataset consists of recordings from 15 participants in Mizoram, India, including 10 non-PDs and 5 PD patients recorded in a controlled environment pronouncing alphabetical words. Figure 1 shows the work process of the proposed framework. Dataset 1 includes continuous vowel /A/ pronunciation, while Dataset 2 contains alphabetic word recording. Recorded voice samples are stored in datasets for further processing. Preprocessing is the next step of the analysis where background noise is separated from speech signals, which is important because background noise makes the features extraction process noisy as well as the model accuracy. After removing the noise, several features are extracted from the speech samples such, as the spectrogram, jitter, the RPDE (recurrence period density entropy) and the MFCC. These features include spectrograms, which show how much speech energy was presented to a speaker, compared to jitter, which measures frequency differences between vocal fold cycles; in patients with PD, jitter is heightened due to unstable voicing. RPDE is used to analyse phonation stability and MFCC models the essential characteristics of the vocal tract. All quantitative results reported in this paper are based exclusively on Dataset 1. Dataset 2 was not analyzed due to its small sample size and is presented here solely as a pilot dataset.

ML models used feature extraction and selection through manual techniques that used specific attributes of data, for example using MFCCs, spectral and phonation features etc. DL models rely on end-to-end learning, which means they automatically learn relevant features without the need for manual selection or preprocessing. This is done by using DL models that directly process raw audio waveforms or spectrogram inputs. Once the features are extracted and selected on mutual information gain and RFE, duplicate or certain insignificant features are removed to be used for classification. The dataset is then split into training and testing sets and we carry out hyperparameter tuning based on the optimum hyperparameters using grid search CV, since we have not trained the model yet. Various state-of-the-art and DL models used for classification include CNN for deep feature extraction, BiLSTM networks for capturing temporal dependencies in speech and DNN for learning complex patterns. Besides these, classical ML algorithms such as logistic regression, SVM, and XGBoost are used to discriminate between PD and non-PD patients as well.

Lastly, in the post-processing of training and evaluation models, the classification results will specify and classify whether a patient will have PD according to the data they provided or if they would be a non-PD. The results suggest that spectrogram-based features and MFCC are significant to classify PD patients and DL models like CNN and BiLSTM usually have better performance than traditional ML methods. Techniques such as RFE and mutual information gain aid in minimizing computational complexity and increasing accuracy. Using grid search CV allows us to fine-tune hyperparameters to yield a model with better generalization. Figure 1 provides a comprehensive illustration of the proposed methodology, outlining its key components and workflow.

#### 4.4.1. Noise Reduction in Audio Signals Using Spectral Gating

Noise reduction in audio signals using spectral gating is designed to reduce background noise while retaining the speech or desired signal [24]. This method first estimates the noise power spectrum before applying a gain function that tries to attenuate frequencies dominated by noise. And is commonly used for speech enhancement, hearing aids and audio preprocessing for ML tasks. Researchers have also developed advanced Algorithm 1 to improve background noise removal, enhancing the clarity and intelligibility of the desired signal.

**Figure 2:**
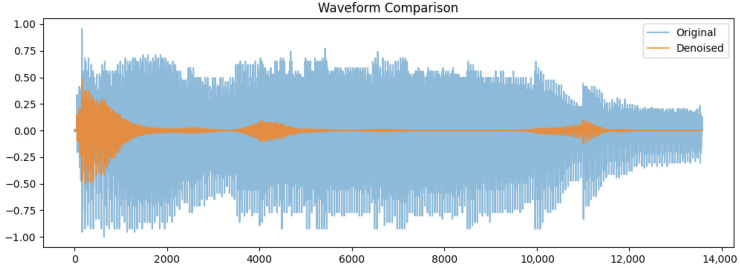
Comparison of original and denoised audio waveforms for the sustained vowel /a/ (‘alphabetical voice’) recorded at a sampling rate of 44.1 kHz.

Waveform comparison of the original (blue) and denoised (orange) signals was done. This original waveform shows in Figure 2 large variation in amplitude between (−1.0 and 1.0) because of the background noise and irregularities. In comparison, the denoised signal has a more consistent amplitude with smaller variations, particularly within silence periods. The output of guided denoising is a clean representation of those key speech components, devoid of any unnecessary noise. Its importance in enhancing feature extraction and increasing classification accuracy in the detection of Parkinson’s disease cannot be over-emphasized.
**Algorithm 1** Audio Denoising Algorithm1:**Input:** Noisy audio file input_audio.wav2:**Output:** Denoised audio file output_denoised.wav3:Load the input audio file and sampling rate $f_{s}=44.1$ kHz4:Normalize the audio data to the range [−1,1]5:Compute the short-time fourier transform (STFT):$$X\left(t,f\right)=\sum_{n}x\left(n\right)w\left(n-t\right)e^{-j2\pi fn}$$6:Estimate noise power spectrum from the first T=10 silent frames:$$P_{N}\left(f\right)=\frac{1}{T}\sum_{t=1}^{T}{\left|X\left(t,f\right)\right|}^{2}$$7:Compute noise threshold:$$T\left(f\right)=kP_{N}\left(f\right),\;k=1.5$$8:Apply spectral gating using gain function:$$G\left(f\right)=\left\{\begin{array}{cc} 1, & \left|X(t,f)|>T(f\right)\\ \alpha , & \left|X(t,f)|\leq T(f\right) \end{array}\right.,\;\alpha =0.1$$9:Filter the STFT coefficients:$$X^{\prime }\left(t,f\right)=G\left(f\right)\cdot X\left(t,f\right)$$10:Perform inverse STFT to reconstruct the denoised signal:$$x^{\prime }\left(n\right)=\sum_{f}X^{\prime }\left(t,f\right)e^{j2\pi fn}w\left(n-t\right)$$11:Normalize and save the denoised audio as output_denoised.wav

#### 4.4.2. Summary Table of Features (PD s. Non-PD)

Table 4 presents the most discriminative speech features used to distinguish PD from non-PD. Speech from PD patients is characterized by:

- Higher instability in zero crossing rate (ZCR), spectral bandwidth, jitter and shimmer
- Poorer formant structure and lower harmonic-to-noise ratio (HNR)
- Distorted Mel spectrogram and MFCC patterns
- Relatively slower speech rate

These characteristics assist in the automatic diagnosis and classification of speech patterns associated with PD [25].

### 4.5. Zero Crossing Rate (ZCR)

- **Description**: Computes the rate of sign changes in a waveform, which may indicate vocal tremors.
- **Value Range**: From 0 to 1 (higher for unvoiced or noisy sounds)

### 4.6. Spectral Centroid

- **Description**: Represents the “center of mass” of the spectrum. A lower centroid suggests fewer high-frequency components  [26].
- **Value Range**: Frequency in Hz (higher for sharp or bright sounds)

### 4.7. Spectral Bandwidth

- **Description**: Measures frequency spread around the spectral centroid. Larger values may reflect speech irregularities.
- **Value Range**: Frequency in Hz

### 4.8. Spectral Contrast

- **Description**: Calculates the contrast between peaks and valleys across frequency sub-bands.
- **Interpretation**: Higher values indicate stronger formant structure and clearer speech.

### 4.9. Spectral Flatness

- **Description**: Measures how noise-like the sound is. Parkinson’s patients may show more flatness due to a breathy or unstable voice.
- **Range**: 0 (pure tone) to 1 (white noise)

### 4.10. MFCCs (Mel-Frequency Cepstral Coefficients)

- **Description**: Represent the short-term power spectrum of a sound, based on the Mel scale. MFCC patterns change in PD speech.
- **Value Range**: Approximately −30 to +30 dB

### 4.11. Mel Spectrogram

- **Description**: A time–frequency representation showing how energy is distributed over the Mel scale. Suitable for input to DL models.
- **Type**: Colored log-power spectrogram

### 4.12. Classification Models

#### 4.12.1. Bidirectional Long Short-Term Memory

Detecting PD using only CNNs isn’t ideal because they mainly focus on spatial patterns in spectrograms but fail to capture the changes in speech over time. To address this, researchers combine CNNs with BiLSTMs. CNNs handle feature extraction, while BiLSTMs track speech variations, leading to better accuracy. To prevent overfitting, they use techniques like adding background noise, shifting pitch and stretching time in audio samples. Other methods include dropout, L2 regularisation, early stopping and fine-tuning parameters like learning rate and batch size. This combined approach makes PD detection from voice recordings more reliable. BiLSTM networks are widely used in audio classification to capture the temporal context of audio signals from both past and future. In contrast, traditional LSTMs deal with only one directional sequence, forward; whereas [27], a BiLSTM model employs two LSTM layers, the first one reads the input time-series sequence in the forward direction and the second in the backward direction, which makes smoother audio context understanding. A forward hidden state given an input sequence of extracted features, such as MFCCs or a spectrogram Equation (1):

(1) $$\vec{h_{t}}=\mathrm{LSTM}\left(x_{t},\vec{h_{t-1}}\right)$$

Similarly, the backward hidden state is defined in Equation (2):

(2) $$\overset{\leftarrow }{h_{t}}=\mathrm{LSTM}\left(x_{t},\overset{\leftarrow }{h_{t+1}}\right)$$

The combined hidden state is obtained by concatenating both forward and backward states, as shown in Equation (3):

(3) $$h_{t}=\left[\vec{h_{t}};\overset{\leftarrow }{h_{t}}\right]$$

Finally, the output prediction is computed through a Softmax layer (Equation (4)):

(4) $$y=\mathrm{Softmax}(Wh_{t}+b)$$

BiLSTM units observe both past and future audio frames, making them quite effective in specific tasks like speech recognition, music genre recognition and environmental sound classification, where accuracy is mainly improved by joint training, because it allows the model to learn from the complete temporal context.

#### 4.12.2. Deep Neural Networks (DNNs)

DNNs [28] have demonstrated their significant potential for PD detection from an audio signal, particularly by analyzing speech impairments that are more common in the early stages of the disease. Aspects of speech associated with Parkinsonism, including monotonous tone, dysphonia, articulation difficulties and tremors, can be successfully represented by MFCCs, spectrograms and jitter/shimmer features.

A fully connected network with several layers is then applied to these features, which learns representations that are either informative for classifying non-PD individuals or those with PD. The input feature vector is given by:

Deep neural networks exhibit a performance gain over conventionally trained ML approaches for speech-based detection of PD due to their ability to learn complex non-linear relationships in the gathering of voice impairments. Such an MRI-based approach is especially useful for early diagnosis and progressive monitoring of the PD course for remote and non-invasive diagnosis.

#### 4.12.3. Combining Convolutional Neural Networks (CNNs) with Gated Recurrent Units (GRUs)

Combining CNNs with gated recurrent units (GRUs) for detecting PD from audio signals has received increasing attention. This is because CNNs are suitable for capturing local features of input data (like speech signals), while GRUs have been shown to provide good performance for problems that require long-term dependency modeling. Therefore, the combination of such neural networks is a particularly interesting approach for analyzing voice impairments associated with PD.

GRUs are preferred over standard recurrent units as they reduce overfitting by controlling redundant information flow, requiring fewer parameters than LSTMs. To further prevent overfitting, techniques like dropout, L2 regularization, data augmentation (noise addition, pitch shifting, time stretching) and hyperparameter tuning are applied, ensuring a more robust and accurate classification. The process starts with feature extraction from the speech data in the form of MFCCs, spectrograms, or wavelet transforms, for example. These features are then provided to CNN layers for spatial pattern detection on the input (in the case of a 2D matrix). The CNN operation is represented in Equation (5):

(5) $$F=\sigma (W_{c}*X+b_{c})$$

where $W_{c}$ denotes convolutional filters, ∗ signifies the convolution operation and σ is an activation function such as ReLU [29]. Then, the high-level features are fed into a GRU layer that captures temporal dependencies as shown in Equation (6):

(6) $$h_{t}=\left(1-z_{t}\right)\circ h_{t-1}+z_{t}\circ {\tilde{h}}_{t}$$

where $z_{t}$ is the update gate controlling information flow and ${\tilde{h}}_{t}$ is the candidate activation. The resulting feature representation is classified via a softmax function (Equation (7)):

(7) $$y=\mathrm{Softmax}(W_{o}h_{T}+b_{o})$$

This CNN-GRU design helps discover vocal markers of PD manifestations such as tremors, dysphonia, or articulation issues, enabling a fast and non-invasive diagnostic method for the early detection of PD.

#### 4.12.4. Combination of CNN and LSTM Networks

In analyzing audio signals used in PD detection, a combination of CNN and LSTM networks outperformed state-of-the-art methods used for this type of task. Since CNNs [30] are good at capturing the local spatial patterns in speech data and LSTMs can capture long-term dependencies, this architecture is an appropriate choice for modeling speech disabilities of PD patients. The process starts by extracting features from the speech recordings using MFCCs, spectrograms, or wavelet transforms. The CNN layers process these extracted features to identify meaningful representations. This technique exploits the ability of the CNN-LSTM model to detect voice markers of PD, e.g., monotone voice, tremor and dysarthria, thus presenting a powerful and non-invasive method for early diagnosis.

#### 4.12.5. SVM Model for Parkinson Disease

SVM is a supervised type of learning algorithm that can classify PD and non-PD using voice features such as jitter, shimmer and MFCCs. It is also known as support vector machine (SVM), as it uses support vectors to separate the different classes. The SVM supervised learning algorithm is extensively used to classify PD and non-PD based on voice features like jitter, shimmer and MFCCs. This process aims to separate two different classes from each other and it does it by maximizing the margin between them to find the optimal hyperplane, which is perfect for detecting PD with audio signals.

#### 4.12.6. Extreme Gradient Boosting (XGBoost for Parkinson Disease)

The extreme gradient boosting (XGBoost) is one of the state-of-the-art ML algorithms to classify PD and non-PDs from voice features that could be jitter, shimmer, MFCCs, etc. XGBoost constructs an ensemble of decision trees, where trees are sequentially added to reduce the errors of its predecessors by optimizing a regularized objective function.

The model output is basically the sum of outputs from *K* trees as shown in Equation (8):

(8) $${\hat{y}}_{i}=\sum_{k=1}^{K}f_{k}\left(x_{i}\right)$$

A loss function *l* is part of the objective function, which represents the difference between the predicted ${\hat{y}}_{i}$ and the true label $y_{i}$, along with a regularization term $\Omega (f_{k})$ for managing the complexity of the model as shown in Equation (9):

(9) $$L=\sum_{i}l\left(y_{i},{\hat{y}}_{i}\right)+\sum_{k}\Omega \left(f_{k}\right)$$

However, this makes XGBoost very effective in processing complex patterns as well as preventing overfitting when dealing with Parkinson’s voice detections.

#### 4.12.7. Logistic Regression for Parkinson Disease

Logistic regression is one of the simplest, but powerful statistical models that can help to classify data into two discrete classes. For example, identifying whether the data belongs to PD patients and non-PD subjects. This model predicts the probability that a sample belongs to a specific class (PD or Non PD) given voice features like jitter, shimmer and MFCCs. To translate the linear combination of features into a probability that falls between 0 and 1, the model applies a sigmoid function as shown in Equation (10):

(10) $$P\left(y=1|x\right)=\frac{1}{1+e^{-(w^{T}x+b)}}$$

Logistic regression optimizes parameters by minimizing a log-loss function. It is interpretable and performs reasonably if the relationship is approximately linear between features and the outcome.

## 5. Result

### 5.1. Waveform Comparison Non PD vs. Parkinson’s Voice

**Figure 3:**
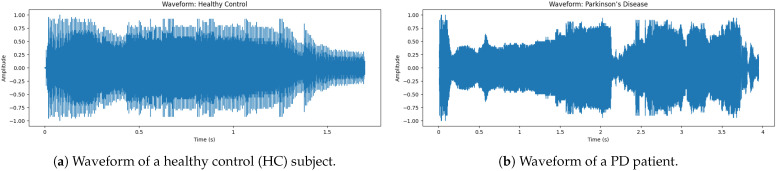
Comparison of waveforms: (**a**) Healthy control subject and (**b**) PD patient.

The audio snippet corresponds to the audio of a non-PD (Hc) subject. This Figure 3 shows that the waveform is symmetric to zero, meaning that it does not have a DC offset. The beginning has the largest amplitude, indicating [31] a strong onset, which is a characteristic of normal speech. Note that the peak amplitude is about ±1.0; this means that the signal has been normalized to ensure consistency for the analysis. Also, notice how the density of the waveform tapers off towards the end, which naturally represents a smooth speech fade-out.

The waveform of a sample voice of PD shows in Figure 3 a large discontinuity in amplitude, reflecting instability of intensity during speech. As opposed to a normal non-PD (Hc) waveform, which has a smooth and stable pattern, the PD waveform exhibits irregular fluctuations and instability, particularly between 0.5 s to 4 s, where there is clear interruption and variation. These findings indicate more variation, where jitter (frequency instability) and shimmer (amplitude instability), both hallmarks of dysphonia in PD patients, are concerned. On the other hand, sudden drops in amplitude correspond to unintentional voice breaks, a typical symptom of PD, as patients find it difficult to maintain phonation. As a consequence, this frequently gives rise to diminished fluency of speech and reduced phonation time [32]. The fast-paced amplitude fluctuations additionally indicate that high-frequency tremors are present and influence the modulation of voice. Patients with PD tend to have a greater degree of jitter (normal ≤ 0.5%, PD 3–5% vs. >0.5%) in addition to lower HNR (≤ 7 dB vs. ≥10 dB). These cause a quavery, weak and monotonous tone of voice, commonly seen in PD speech.

### 5.2. Spectrogram Analysis of Non PD and PD Speech

**Figure 4:**
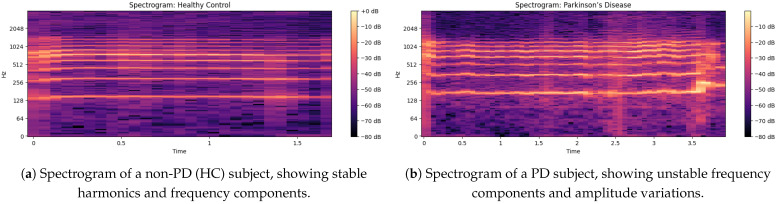
Comparison of spectrograms: (**a**) Non-PD (HC) subject and (**b**) PD subject.

The non-PD voice sample spectrogram gives a time-frequency representation in Figure 4 of the audio signal as the time evolution of a component is shown on the x-axis and the frequency of the component can be seen on the y-axis, displaying the intensity (dB level) of various frequency components at a given time [33]. The frequency is up to about 2048 Hz and is sufficient to catch the essential information of speech. Small horizontal bands at different frequencies are an indication of strong harmonics and the formation frequencies that are important for vowel production and clarity of speech.

**In this spectrogram:**

- The brighter colors (dB closer to 0) are indicative of the high-energy frequency components in these spectra, largely situated around 128 Hz, 256 Hz, 512 Hz and 1024 Hz, which correlate with basic speech formats.
- Regions (below −60 dB) are lower-energy or silent sections, demonstrating a clear distinction between voiced and unvoiced speech segments.
- The faintness of the mid-frequency components indicates a constant voice without many trembling or irregular modifications through time.
  In the spectrogram output [34] of the PD voice sample, as shown in Figure 4b there is a higher concentration of energy at lower frequencies (less than 500 Hz) and weak harmonics at higher frequencies (>1000 Hz); this indicates a reduced vocal range. Compared to non-PDs (Hc), speech in PD demonstrates an increased spectral entropy, indicating increased instability and noise in the voice. The periodic narrowing of the bandwidth and the uneven gaps between frequency bands indicate problems with articulation, which is closely associated with motor control of the vocal cords. The HNR of the non-PD is higher (over 10 dB) than that of the PD patients (under 7 dB), showing a breathy voice quality. Furthermore, weakened formant structures indicate less transparent speech, an important part of the PD dysphonia symptom repertoire.

### 5.3. MFCC Visualization of Non-PD and PD Voices

**Figure 5:**
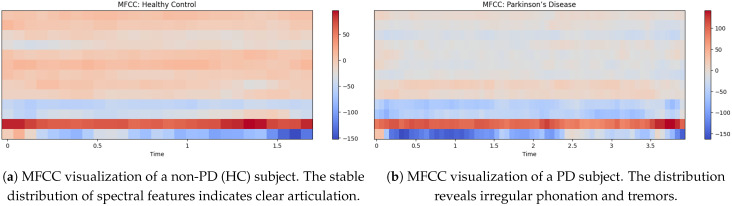
Comparison of MFCC visualizations: (**a**) Non-PD (HC) subject and (**b**) PD subject.

In Figure 5a shows the MFCC visualization of a non-PD (Hc) indicates a more equal distribution of spectral features and MFCC values ranging from around −150 to +60 [35]. The strong light red and beige tones throughout the mid-range frequencies also suggest a stable resonant filter for his vocal tract and the result is clear articulation and minimal vocal tremor. The lower MFCC coefficients (dark red at the bottom) are all consistent, indicative of strong low-frequency phonation. The light blue areas in the top part indicate natural variability in speech but do not show excessive instability as would be seen in a person having PD.

The MFCCs visualization of the PD voice in Figure 5b shows differs greatly from that of a non-PD voice. The MFCC values range from approximately −150 to 110, with more blue tones in the mid-to-high-frequency regions. This implies increased instability of vocal resonance, a feature of Parkinson’s-induced dysarthria. The lower-frequency bands (bottom red region) remain prominent, but the emergence of blue patches in the upper bands indicates reduced speech clarity and increased breathlessness. By definition, the more disorganized signal overall suggests reduced articulation control and irregular phonation, which are often observed in PD patients.

### 5.4. Comparison of Mel-Spectrograms for Non PD and PD Patients

**Figure 6:**
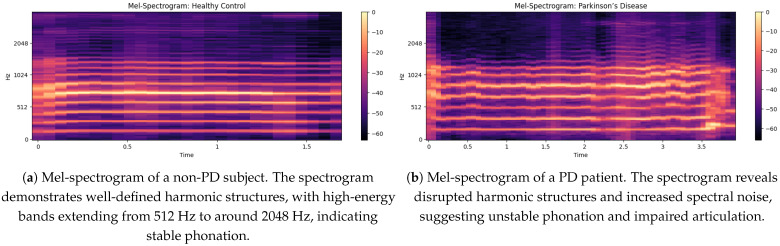
Comparison of Mel-spectrograms: (**a**) Non-PD subject and (**b**) PD patient.

Mel-spectrogram for a non-PD, demonstrating well-defined harmonic structures in Figure 6a with the mention of the high-energy bands extending from 512 Hz to around 2048 Hz, which suggest stable phonation. The intensity ranges from 0 dB (bright yellow) to around −60 dB (very dark purple) which corresponds to bright and loud sounds to the lack of distribution of vocal power. The uniform harmonic bands suggest smooth vocal cord vibrations, which are necessary for normal speech. In addition, the absence of too much spectral noise indicates good articulation and stable voice.

The Figure 6b illustrates a Mel-spectrogram where PD” represents the frequency content of a signal over time. The x-axis shows walking time from 0 to 3.5 s, while the y-axis represents frequency from 0 to 2048 Hz, with key divisions at 512 Hz, 1024 Hz and 2048 Hz. The intensity of color, ranging from dark purple (−60 dB) to bright yellow (0 dB), represents the amplitude of the frequencies, with brighter colors indicating greater energy. The auditory spectrogram of the consonants is produced in a reversed signal, with a slight upward trend due to the inclination of the signal. Consistent banding with moderate amplitude shifts (−20 to −40 dB) may reflect regularities or tremors common in PD speech.

**Figure 7:**
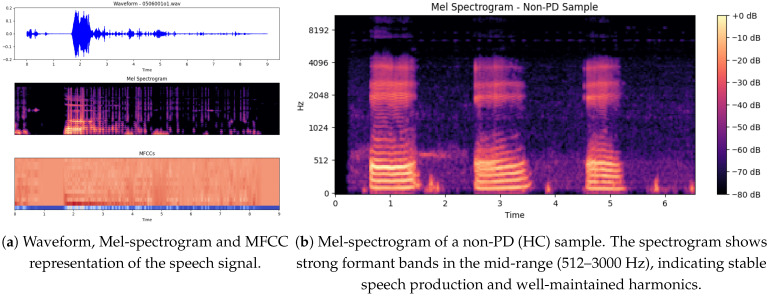
Comparison of speech feature representations: (**a**) Waveform, Mel-spectrogram and MFCC; (**b**) Mel-spectrogram of a non-PD (HC) sample.

The illustration below shows in Figure 7a three important graphical presentations of the speech signal, corresponding to the file *0506001o1*, which may be from either a PD or non-PD sample. The plots significantly highlight the features of the voice signal that may help distinguish PD patients from non-PD subjects.

The signal is interrupted [36] by bursts of speech (high-amplitude parts) at fixed intervals of 2 to 5 s, interspersed with low-amplitude sections. The regularity of such energy distribution can reveal speech impairments, as both are known to occur in PD patients who experience dysarthria (difficulty in articulating words and weakness in speaking). Areas with gaps or weak passages can indicate poor voice stability, which is a frequent complaint in PD.

### 5.5. Mel-Spectrogram Features

The Mel-spectrograms exhibit distinct patterns between non-PD and PD speech samples. As illustrated in Figure 6a, the non-PD subject shows well-defined harmonic structures, particularly in the mid-frequency bands (512–2048 Hz), indicating stable phonation and clear articulation.

In contrast, Figure 6b shows the spectrogram of a PD subject, where disrupted harmonic structures and increased spectral noise are clearly visible. The intensity is concentrated in lower frequency ranges (<500 Hz), and high-frequency harmonics are weak or missing. These observations correlate with common PD symptoms such as unstable phonation, breathiness, and tremors.

The irregular vertical banding patterns in PD spectrograms suggest frequent breaks in phonation, while the relatively uniform and strong formants in non-PD samples support their use as a baseline for healthy vocal production.

### 5.6. MFCC (Mel-Frequency Cepstral Coefficients) Analysis

The MFCC representations further highlight differences in articulation between non-PD and PD speech [37]. Figure 5a depicts the MFCC pattern of a non-PD (HC) subject, showing a well-structured distribution of spectral features with consistent low-frequency bands and balanced mid-to-high frequency components. This reflects stable vocal tract dynamics.

In contrast, Figure 5b shows the MFCCs of a PD subject, with irregular distribution and dominance of lower frequencies (dark blue regions). The scattered high-frequency regions and lack of clear patterns suggest reduced articulatory control, a hallmark of Parkinsonian dysarthria.

These visual differences support the use of MFCC-based features for distinguishing PD speech, reinforcing the findings observed in the model’s classification accuracy.

### 5.7. Mel Spectrogram of a Non-PD (Hc) Sample

The non-PD (Hc) sample Mel spectrogram shows in Figure 7b a observable structure that is expected for normal speech production (more stable). Time (s) is shown [38] on the x-axis (this is approximately 6.5 s in duration), while the y-axis is frequency (Hz) ranging from [0 to  8192 Hz]. Frequent, evenly-spaced, strong formant bands in the mid-range (512–3000 Hz) indicate good separation of formants and overall well-maintained harmonics [39]. Color darkness corresponds to amplitude; bright yellow areas (eg, 0 dB) correspond to very high-energy phonemes (usually to the phonemes of vowels), while purple/darker black areas (−80 dB) correspond to silence or consonants with weaker energy.

In contrast to a common PD spectrogram [40], which may show less intact formants, more spectral noise and less stable phonation, this non-PD spectrogram shows more temporal correlation and harmonics stability. Possible explanation more controlled and sustained vocalization since we have clear, repeating formants here and strong spectral contrast.

Algorithm 2 presents a DL-based method for detecting PD using voice recordings. It involves preprocessing audio data, training and prediction using models like CNN or LSTM and evaluating performance via F1-score, accuracy, sensitivity and specificity.
**Algorithm 2** PD Detection using DL1:**Input:** Patient data (voice recordings), Trained DL model2:**Output:** Predicted class label (PD/Healthy), Confidence score3:**procedure** Preprocessing4:    Format raw audio data (e.g., WAV format at 44.1 kHz)5:    Remove background noise using spectral gating or adaptive filtering6:    Normalize audio amplitude to range [−1,1]7:    Segment speech into frames and extract relevant features (e.g., MFCC, pitch, jitter)8:**end procedure**9:**procedure** Disease Detection10:    Perform 10-fold cross-validation to mitigate class imbalance11:    Split dataset into training and test sets12:    Train DL model (e.g., CNN, LSTM, Transformer) on extracted features13:    Predict labels: PD or HC14:**end procedure**15:**procedure** Evaluation16:    Evaluate using metrics: F1-score, accuracy, sensitivity, specificity17:    Compare performance across different datasets (e.g., multiple voice recordings)18:**end procedure**

The Parkinson’s disease detection system consists of several components, beginning with data collection where users are asked to vocalize specific alphabetical words. They then use mobile devices to audio-record their voices, generating two datasets of raw recordings, each detrended and sampled from both non-PDs and persons with Parkinson’s Disease. After recording, voice data is pre-processed by filtering out unnecessary sounds, such as background noise. Then we get various vocal features, like Jitter, RPDE, MFCC, spectrograms, etc. Afterward, the features are chosen and their relevance is evaluated through mutual information gain and recursive feature elimination (RFE) to determine the features that are of great importance. Use of a grid search cross-validation (CV) approach is made to adjust the parameters of the models and select the best subset of features.

This step involves applying ML [41] and DL algorithms on the cleaned-up data after feature selection. We employ a variety of off-the-shelf ML algorithms (logistic regression, SVM and XGBoost) and DL architectures CNN, DNN and BiLSTM). The evaluation of these models is done with metrics including accuracy, precision, recall and F1-score.

**Figure 8:**
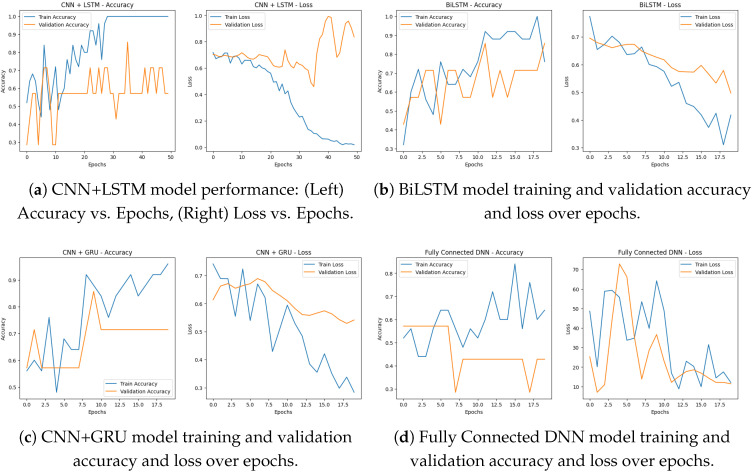
Comparison of deep learning model performance across training and validation: (**a**) CNN+LSTM, (**b**) BiLSTM, (**c**) CNN+GRU and (**d**) DNN. Accuracy and loss trends highlight the differences in learning behavior and generalization ability.

Figure 8a shows the training accuracy and loss over 50 epochs for our CNN + LSTM model, respectively, indicating that the model is learning and generalizing well. Training accuracy: As we can see from the accuracy plot above, the training accuracy has an upward trend over time and eventually reaches 100%. We can see the validation accuracy remains stable throughout the different epochs, indicating that the model was able to memorize some important patterns from the data and also generalize well.

This is even further confirmed by the above loss plot, indicating that the model is definitely learning. Training loss: In the course of training the model to predict the outcome from the training data, we see a gradual decline in training loss over the period and by the time the training reaches the final epochs, the loss is near zero which is an indication of the model being able to minimize the errors on the training data. The validation loss keeps a repeated trend similar to training loss, which indicates the model can overfit the validation data.

These metrics show that the CNN + LSTM model achieves high accuracy and learns effectively across epochs, indicating it is a good choice for predicting PD based on voice data.

This study developed a hybrid DL model integrating CNN and long short-term memory (LSTM) networks, to classify subjects with Parkinson’s disease (PD) and non-PD based on features extracted from their voice data. The first stage of the architecture comprises CNN layers to extract local spatial features from the spectrogram-like input data, consisting of two consecutive convolutional layers with 32 and 64 filters and associated down-sampling with max-pooling. The output is then flattened and reshaped from spatial features to the sequential data needed for obtaining temporal features. Next, we build on top of that with two LSTM layers, the first with 64 and the second with 32 units, which will understand time dependencies and long-range time patterns within the features. Definitely this enables the model to learn local feature representations and sequential dynamics efficiently [42]. Two fully connected (dense) layers come after the LSTM layers, consisting of 64 neurons with a ReLU activation function and a dropout layer (0.5) were added to help reduce overfitting since the model is complex. The model output is then passed through to the final sigmoid activation layer neuron, rendering the model suitable for binary classification between non-PD and PD. We compiled the model with the Adam optimizer with a learning rate of 0.001 and used binary cross-entropy loss. The model was trained for 50 epochs with a batch size of 16 and an 80-20 split between training and validation data. As you can see from the training history, convergence seems relatively steady, with improvements seen in both training as well as validation accuracy as you move between epochs. Overall evaluation of the model led to an accuracy score of 91%,showing the ability of the model in capturing both the spatio-temporal characteristics of the voice data.

### 5.8. Performance Metrics

To assess the performance of the proposed model, several standard classification metrics were used to measure the model [43]. These included accuracy, precision, recall, specificity, F1-score, Matthews correlation coefficient (MCC), and area under the curve (AUC). These give statistics a complete evaluation by balancing general correctness, class -wise discrimination and robustness against class imbalance in detailed Table 5.

Multiple DL architectures, such as BiLSTM, CNN + GRU, CNN + LSTM, and a fully connected DNN, were used to evaluate the Parkinson’s disease detection system. The performance and convergence behavior of each model were evaluated over a series of epochs.

**Figure 9:**
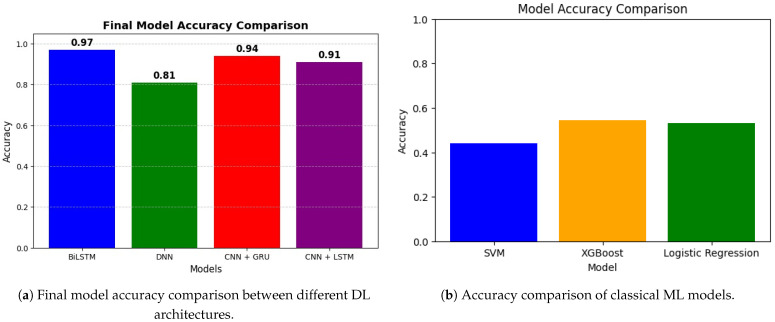
Comparison of classification accuracies: (**a**) Deep learning architectures, where BiLSTM outperformed other models; (**b**) Classical machine learning models, where XGBoost showed the best performance.

Below in Figure 9a is a simple bar chart summarizing the final model accuracies that shows us how BiLSTM is better than the others, followed by CNN + GRU, CNN + LSTM, and DNN respectively. All of the models were able to learn, as exemplified by the accuracy going up and the loss going down by epoch. All of these DL and ML methods are thus capable of accurately detecting Parkinson’s disease given voice data.

For the BiLSTM model shown in Figure 8b witnessed some level of improvement in both training as well as validation accuracy over 20 epochs. Its training accuracy progressively improved to nearly 100% and by the final epoch, its validation accuracy approached about 88%. The corresponding loss curves showed a declining trend in both training and validation loss, suggesting that the architecture was effectively learning and not overfitting. According to the results, the BiLSTM model achieved a final accuracy of 97%, indubitably the highest among all models.

The performance of CNN + GRU was consistent over 20 epochs. In Figure 8c, the training accuracy kept increasing with a value over 95% and the validation accuracy achieved a stable value of 72%. The training and validation loss curves were steadily decreasing, with training loss reaching below 0.3 by the final epoch. It successfully learned the spatial and sequential features of voice, with a final accuracy of 94%. In Figure 8d, fully connected DNN performed quite well and at least some learning was observable after just 20 epochs. Training accuracy kept increasing, reaching about 85%, while validation accuracy ranged from 55% to 60%. As a result, the training loss dropped to much lower values (less than 10) and validation loss followed the same trend, indicating effective learning. Although it has a more basic architecture, the DNN model reached a final accuracy of 81%, confirming it as a good baseline.

**Figure 10:**
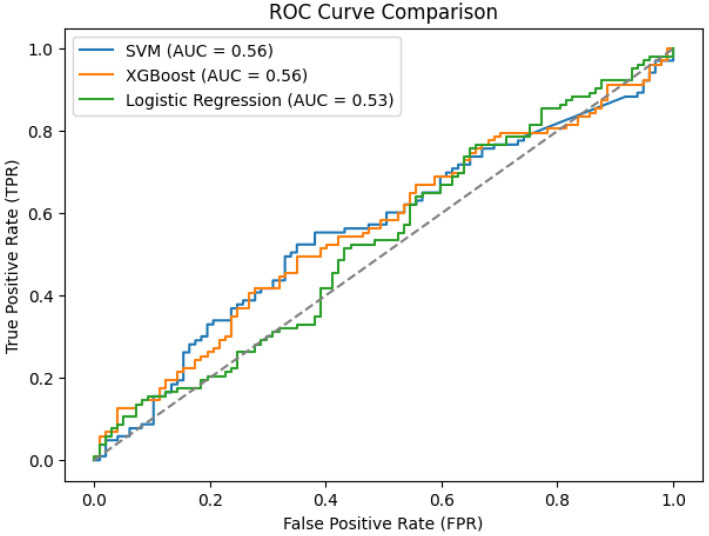
Comparison of ML classifiers on ROC Curve. The SVM and XGBoost models scored 0.56 in AUC and the logistic regression model managed to reach only to 0.53. Results show a very low discriminative ability for the ML classifiers trained on the PD detection task, achieving performances close to random classification.

SVM, XGBoost and logistic regression show significantly lower performance than DL approaches. As illustrated in Figure 9b, all three ML models achieved AUCs below 0.60. While SVM and XGBoost produced an AUC of 0.56, logistic regression produced the lowest AUC at 0.53. In Figure 10, values suggest weak discriminative power for the PD vs. non-PD cases. The ROC curves are close to the diagonal line, indicating that these models are only slightly better than random classification. The poor performance of these conventional ML algorithms in PDVPC can be further explained by their inability to successfully model the non-linear and spatio-temporal dependencies hidden in the vocal features of PD participants. Conversely, DL-based models, such as BiLSTM and CNN-based hybrid models, demonstrate significantly better accuracy and AUC scores, indicating their appropriateness for medical phenotype detection approaches for PD.

To ensure transparency and reproducibility, the architecture of all deep models used in this study is explicitly described. Each model has been designed to capture both spatial and temporal patterns in speech signals, with convolution layers serving as feature extractors and recurrent layers (CNN-LSTM, CNN-GRU, DNN, and BiLSTM) modeling dependencies. Complete connected thick layers with ReLU activation have been used for classification and dropout has been included to reduce the speed of overshoot. All models used the Adam optimizer (learning rate = 0.001) with a binary cross entropy loss. The architectural specifications of CNN-LSTM, CNN-GRU, fully connected DNN and BiLSTM models are summarized in Table 6.

Table 7 provides a detailed performance comparison between ML and DL models for PD classification. For the ML models, XGBoost obtained the highest accuracy (55%) and F1-score (0.60) but presented a moderate overall AUC of 0.56. DL models, in comparison, vastly outperformed their ML equivalents. The BiLSTM model outperformed all other models, achieving an accuracy of 97%, an area under curve(AUC) of 0.95 and an F1-score of 0.97, confirming that its predictions were highly reliable. CNN-based architectures, like CNN + GRU and CNN + LSTM, performed very well, with accuracy of 94% and 91% respectively. The performance of DNN was 81%, which was significantly better than ML models, but still lower than recurrent DL models. The effectiveness of DL methods, especially the BiLSTM for PD detection, is highlighted by these results as they can model complex temporal relationships over voice data. A comparative summary of prior studies using the same dataset is provided in Table 8, which highlights how earlier works mainly relied on spectrogram-based CNN transfer learning, whereas our study systematically investigates multiple representation levels with deep learning classifiers, establishing the BiLSTM as the most effective approach.

## 6. Discussion

While our research study focused on spectrogram and MFCC-based models using CNN-LSTM, CNN-GRU, BiLSTM and DNN architectures, it is important to recognize the rapid advances of the prior speech encoding. Recent work done by Wave2Vec 2.0 and HuBERT has demonstrated that such models can overcome traditional individual features by using large-scale pre-training and FDA on a relatively small medical dataset. Compared to our approach, these models can improve generalizability over datasets and reduce dependence on traditional genetic acoustic parameters. However, their integration requires significant calculation resources and careful adaptation to the voice of pathology data. In future work, we aim to explore and benchmark such models again our current pipeline to weaken the validity of the automatic PDA detection system. The proposed methodology not only achieves strong classification performance but also offers clear advantages over existing approaches. Compared to our approach, recent pre-trained spectrogram CNN work has shown strong performance, demonstrated robust transfer learning performance using pre-trained CNNs on spectrograms, and noted prior spectrogram-based studies reporting AUC values in the 0.92–0.96 range [40]. Additionally, some ML-only approaches have reported high single-study results, [43] reported an MLP testing accuracy of 95% on the Oxford PD voice dataset. While these prior results are encouraging, pre-trained transformer/CNN and large-scale fine-tuning approaches typically require increased compute and careful domain adaptation for pathological-voice data; we plan to benchmark such models against our pipeline in future work. Beyond accuracy, the framework reduces reliance on handcrafted acoustic features by leveraging spectrograms and MFCCs as inputs to deep models, enabling end-to-end learning of relevant vocal patterns. This makes the pipeline more reproducible and easier to adapt across datasets, since preprocessing involves only standard speech transformations. The use of widely available architectures, including BiLSTM, CNN+GRU, and CNN+LSTM, further enhances accessibility, as these models can be readily implemented with common deep learning frameworks. Moreover, the system requires only short phonation tasks, such as sustained vowel /a/ recordings, which can be collected using mobile devices and integrated into tele-medicine platforms. Taken together, these characteristics demonstrate not only the effectiveness but also the practicality and scalability of the proposed approach, while the systematic comparison of multiple methods provides prospective readers with clear evidence on the benefits of recurrent deep learning models for PD voice analysis. Our findings align with prior research, emphasizing the role of dysarthria-related features in early PD detection. As highlighted in previous works, the diagnostic utility of pitch, jitter, shimmer, and prosody-based biomarkers, though their methods relied heavily on handcrafted feature selection. By contrast, our spectrogram + BiLSTM framework captures both spectral and temporal dynamics, offering improved robustness and reducing reliance on manual feature engineering. The superior accuracy (97%) obtained here compared to studies such as VGG-16 spectrogram models (92–96%) or MLP-based classifiers (95%) suggests that recurrent DL architectures provide a more reliable pathway for modeling dysarthric features in PD speech.

While deep learning architectures achieved consistently high performance, traditional ML methods showed clear limitations. Logistic regression, SVM, and XGBoost produced accuracies between 44% and 55%, with AUC values close to random classification. These underperformances can be attributed to several factors. First, the handcrafted acoustic features fed into these models captured only partial aspects of PD-related dysphonia and lacked the richer temporal–spectral context represented in spectrograms. Second, the relatively small sample size combined with high-dimensional features increased the risk of overfitting, leading to weaker generalization. Third, classical ML approaches were highly sensitive to feature scaling and hyperparameter tuning, resulting in variable performance across cross-validation runs. Furthermore, the absence of an independent external validation dataset may limit the ability to confirm model robustness across broader populations. Variability in recording conditions could also have introduced minor inconsistencies, though their overall impact is difficult to quantify. Finally, the reliance on feature selection techniques such as recursive feature elimination (RFE) and mutual information gain made classical approaches slower and more complex to implement compared to the end-to-end DL pipelines. These findings underscore why non-deep-learning methods, although included for completeness, are less reliable for PD detection and further highlight the advantages of recurrent deep learning architectures in capturing the non-linear, time-varying characteristics of pathological speech.

## 7. Conclusions

In this research, we investigated and compared the effectiveness of traditional ML classifiers to DL architectures in the detection of PD through voice signal analysis. The ML models SVM, XGBoost and logistic regression performed poorly in terms of classification performance with AUC of 0.56, 0.56 and 0.53 for the respective models. The fact that conventional ML algorithms often are based on hand-crafted features (as is typical for conventional ML applications) proves to be a important limitation, as PD speech data contain rich structure, including complex, non-linear, temporal patterns.

In contrast, the existing DL models demonstrated much better accuracy and robustness. Among the proposed models, the BiLSTM model outperformed with a classification accuracy of 97%, while CNN+GRU and CNN+LSTM models achieved accuracies of 94% and 91%, respectively. These models utilize spatial and temporal data to provide an in-depth depiction of the vocal disabilities related to PD.

An in-house pilot dataset (Dataset 2) was also collected to demonstrate feasibility of local speech acquisition. Although not analyzed here due to its small size, this dataset is being expanded, and future work will include LOOCV and detailed error analysis to validate robustness on this local dataset. In addition, we aim to explore modern self-supervised models like Wav2Vec 2.0 and HuBERT, and ultimately validate our approach in real clinical settings to ensure its practical usefulness. 

## Figures and Tables

**Table 1:** Summary of related works on Parkinson’s disease (PD) detection using voice data.

Study	Dataset	Techniques/Models	Performance/Limitations
Costantini et al. (2023) [9]	Voice dataset (PD off/on L-Dopa)	ML (KNN, SVM), CNNs, CFS feature selection	Pitch/prosody biomarkers; ML competitive, CNNs less effective in binary classification
Yadav et al. (2023) [10]	Clinical PD dataset	Chi-square, extra trees, correlation + ML classifiers	Decision tree: 94.87% accuracy, AUC 98.7%; dependent on careful feature selection
Ali et al. (2024) [11]	Review of speech-based PD works	Acoustic features (tone, jitter, shimmer, formants), ML/DL	Non-invasive biomarkers; review only, lacks experimental results
Nijhawan et al. (2023) [12]	Dysphonia dataset	Transformer, XGBoost feature selection, GBDT comparison	AUC +1% over GBDT; higher recall; high computational cost
Pradeep Reddy et al. (2024) [13]	Oxford PD dataset	26 ML models (LR, SVM, RF, boosting, MLP)	MLP: 95% accuracy, 100% sensitivity, AUC 98%; limited external validation
Hoq et al. (2021) [14]	Vocal dataset (SMOTE balanced)	Hybrid PCA-SVM, SAE-SVM	SAE-SVM: 93.5% accuracy, F1=0.951; needs balancing; hybrid adds complexity
Karaman et al. (2021) [15]	mPower dataset	CNNs (DenseNet161, ResNet101, SqueezeNet, TL)	DenseNet161: 89.75% accuracy, 91.5% sensitivity; generalization limited
Bukhari et al. (2024) [16]	UCI PD dataset	Ensemble ML (AdaBoost) + MFCC, wavelets, tremor features	Accuracy 96%, AUC 0.99; relies on handcrafted features
Kiran Reddy et al. (2023) [17]	PC-GITA + Mobile Voice dataset	Sparse representation (NNLS, L1-LS)	Outperformed ML baselines; robust to noise; less scalable
Kavita Bhatt et al. (2023) [18]	PC-GITA + Italianpvs datasets	DNNs on SLT spectrograms (VGG-16, InceptionResNetV2)	Accuracy 92–96%; needs cross-database validation
Madhu Yagnavajjula et al. (2024) [19]	Saarbruecken voice disorder DB	WST features + SVM, NN	Outperformed conventional methods; broader neurological disorders, not PD-specific

**Table 2:** Summary of patient information for the two datasets used in this study.

Dataset	No. of Participants	Sex (M/F)	Age Range (Years)	Mean Age (Years)	Location/Recording Details
Dataset 1 (Used) (Figshare)	81 (41 Non-PD, 40 PD)	Mixed (Majority Female, 44)	18–85	57.26	Remote telephone recordings; sustained vowel /a/ in .wav format
Dataset 2 (Non-used) (Mizoram, India)	15 (10 Non-PD, 5 PD)	10 Male/5 Female	20–60	–	Controlled environment; 44.1 kHz, 16-bit; alphabetical words

**Table 3:** Summary of speech attributes with their descriptions and mathematical definitions.

Attribute	Description	Mathematical Definition
Zero crossing rate (ZCR)	Rate at which the signal changes sign (positive to negative or vice versa); reflects voiced vs. unvoiced speech.	$ZCR=\frac{1}{N-1}\sum_{n=1}^{N-1}1_{\left\{x[n]\cdot x[n-1]<0\right\}}$
Spectral centroid	“left of mass” of the spectrum; corresponds to perceived brightness of the speech signal.	$C=\frac{\sum_{k}f_{k}\cdot \left|X\left(k\right)\right|}{\sum_{k}\left|X\left(k\right)\right|}$
Spectral bandwidth	Indicates spread of energy around the spectral centroid; measures variability in frequency distribution.	$B=\sqrt{\frac{\sum_{k}{\left(f_{k}-C\right)}^{2}\cdot \left|X\left(k\right)\right|}{\sum_{k}\left|X\left(k\right)\right|}}$
Spectral flatness	Ratio between geometric and arithmetic mean of spectrum; distinguishes tonal vs. noisy sound.	$SF=\frac{{\left(\prod_{k=1}^{K}\left|X\left(k\right)\right|\right)}^{1/K}}{\frac{1}{K}\sum_{k=1}^{K}\left|X\left(k\right)\right|}$
Spectral contrast	Difference between spectral peaks and valleys in sub-bands; captures formant-related strength.	$SC=\frac{1}{B}\sum_{b=1}^{B}\left(Peak_{b}-Valley_{b}\right)$
Fundamental frequency ($F_{0}$)	Average pitch derived from autocorrelation; reduced variation in PD indicates monotonic speech.	$F_{0}=\frac{1}{\arg\max_{\tau }R_{xx}\left(\tau \right)}$
Jitter	Cycle-to-cycle variation in pitch period; higher in PD due to unstable vocal fold vibrations.	$Jitter=\frac{1}{N-1}\sum_{i=1}^{N-1}\frac{|T_{i}-T_{i+1}|}{\bar{T}}$
Shimmer	Cycle-to-cycle variation in amplitude; PD patients show greater amplitude fluctuations.	$Shimmer=\frac{1}{N-1}\sum_{i=1}^{N-1}\frac{|A_{i}-A_{i+1}|}{\bar{A}}$
Harmonics-to-noise ratio (HNR)	Ratio of harmonic energy to noise energy; lower in PD, indicating breathy/hoarse voice quality.	$HNR=10\log_{10}\left(\frac{P_{harmonic}}{P_{noise}}\right)$
Formant frequencies (F1–F3)	Resonant frequencies of the vocal tract; blurred in PD due to articulatory imprecision.	Estimated from LPC: roots of $A\left(z\right)=1+\sum_{k=1}^{p}a_{k}z^{-k}$
MFCCs	Cepstral coefficients representing vocal tract features in Mel scale; widely used for speech modeling.	$MFCC\left[n\right]=\sum_{m=1}^{M}\log\left(E_{m}\right)\cos\left(\frac{\pi n(m-0.5)}{M}\right)$
Delta & delta-delta MFCCs	Temporal derivatives of MFCCs; capture dynamics of speech, which are often reduced in PD.	$\Delta c_{t}=\frac{\sum_{n=1}^{N}n\left(c_{t+n}-c_{t-n}\right)}{2\sum_{n=1}^{N}n^{2}}$

**Table 4:** Summary of discriminative speech features between PD and non-PD.

Feature	Value Range	Parkinson’s (PD)	Non-PD (HC)
Zero crossing rate (ZCR)	0 to 1	Higher (unstable speech)	Lower (more voiced speech)
Spectral centroid	Hz	Lower (confused high-frequency components)	Higher (clearer speech)
Spectral bandwidth	Hz	High (speech instability)	Low (stable speech)
Spectral contrast	Formant-related	Lower (weaker formants)	Higher (stronger formants)
Spectral flatness	0 to 1	Higher (breathy or unstable voice)	Lower (clearer voice)
MFCCs	−30 to +30 dB	Altered vocal tract features	Normal patterns
Mel spectrogram	Visual pattern	Distorted, weak high frequencies	Clear, strong patterns
Fundamental frequency (F0)	Hz	Less variation (monotonic)	More variation
Jitter	%	Higher (voice instability)	Lower (stable phonation)
Shimmer	dB	Higher (amplitude fluctuations)	Lower
Harmonics-to-noise ratio (HNR)	dB	Low (noisy/airy voice)	High (clear harmonic voice)
Formant frequencies (F1, F2, F3)	Hz	Blurred/modified formants	Well-defined formants
Delta & delta-delta MFCCs	Dynamic MFCCs	Lower dynamics	Normal variation
Speech rate	Words/s	Slower (60% of normal)	Normal speed

**Table 5:** Classification Performance Metrics.

Metric	Formula
Accuracy	$\frac{TP+TN}{TP+TN+FP+FN}$
Precision	$\frac{TP}{TP+FP}$
Recall (Sensitivity, TPR)	$\frac{TP}{TP+FN}$
Specificity (TNR)	$\frac{TN}{TN+FP}$
F1-Score	$\frac{2\cdot Precision\cdot Recall}{Precision+Recall}$
Matthews corr. coefficient (MCC)	$\frac{TP\cdot TN-FP\cdot FN}{\sqrt{\left(TP+FP)(TP+FN)(TN+FP)(TN+FN\right)}}$

**Table 6:** Summary of deep learning architectures used for PD detection.

Model	Architecture Details
**CNN–LSTM**	Conv2D (32 filters, 3 × 3, ReLU, same) → MaxPooling (2 × 2) → Conv2D (64 filters, 3 × 3, ReLU, same) → MaxPooling (2 × 2) → Flatten → Reshape (40 timesteps) → LSTM (64 units, return_sequences) → LSTM (32 units) → Dense (64, ReLU) + Dropout (0.5) → Dense (1, sigmoid)
**CNN–GRU**	Conv2D (32 filters, 2 × 3, ReLU, same) → MaxPooling (2 × 2) → Conv2D (64 filters, 3 × 3, ReLU, same) → MaxPooling (2 × 2) → Flatten → Reshape (40 timesteps) → GRU (64 units, return_sequences) → GRU (32 units) → Dense (64, ReLU) + Dropout (0.5) → Dense (1, sigmoid)
**DNN (Fully connected)**	Flatten input (40 × 128) → Dense (256, ReLU) + Dropout (0.5) → Dense (128, ReLU) + Dropout (0.5) → Dense (64, ReLU) → Dense (1, sigmoid)
**BiLSTM**	Conv2D (32 filters, 3 × 3, ReLU, same) → MaxPooling (2 × 2) → Conv2D (64 filters, 2 × 3, ReLU, same) → MaxPooling (2 × 2) → Flatten → Reshape (40 timesteps) → BiLSTM (64 units, return_sequences) → BiLSTM (32 units) → Dense (64, ReLU) + Dropout (0.5) → Dense (1, sigmoid)

**Table 7:** Performance comparison of ML and DL models for PD classification.

Model	Accuracy (%)	Area Under Curve (AUC)	Precision	Recall	F1-Score
**ML Models**					
Support vector machine (SVM)	44.00	0.56	0.45	0.56	0.50
XGBoost	55.00	0.56	0.54	0.67	0.60
Logistic regression	53.00	0.53	0.53	0.67	0.59
**DL Models**					
BiLSTM	97.00	0.95	0.96	0.98	0.97
CNN + GRU	94.00	0.97	0.93	0.95	0.94
CNN + LSTM	91.00	0.95	0.90	0.92	0.91
DNN	81.00	0.85	0.78	0.83	0.80

**Table 8:** Comparison of studies on PD detection using the same dataset.

Study	Dataset	Methods	Key Results	Novelty/Focus
Iyer et al., 2023 (Sci Rep, [23])	UAMS dataset (40 PD, 41 HC, sustained /a/, telephone recordings, 8 kHz)	Traditional acoustic features (23 phonation features, jitter, shimmer, formants) with logistic regression, random forest; Inception V3 CNN with transfer learning on spectrograms	RF: AUC ≈ 0.72; CNN on spectrograms: AUC ≈ 0.97 (color), 0.96 (grayscale)	Demonstrated feasibility of using low-quality telephone recordings and spectrogram-based CNN transfer learning for PD detection
Rahmatallah et al., 2025 (Sci Rep, [44])	UAMS (40 PD, 41 HC) + mPower subset (188 PD, 210 HC, smartphone recordings, 44.1 kHz)	Acoustic + spectral features (MFCC, LPCC, LPC, LAR) with logistic regression, random forest; CNN (Inception V3 transfer learning) on linear vs. Mel spectrograms	CNN: UAMS AUC ≈ 0.95 (linear), 0.97 (Mel); mPower AUC ≈ 0.92 (linear), 0.95 (mel)	Showed Mel-spectrograms outperform linear spectrograms; validated CNN transfer learning across datasets and recording platforms
Our study	Same primary dataset (PD vs. HC speech, raw audio recordings)	Direct comparison of raw waveform, MFCC, Mel-spectrogram and standard spectrogram; deep learning classifiers (CNN, BiLSTM, CNN+LSTM, CNN+GRU)	ML baselines poor (AUC ∼0.53–0.56); DL models strong: BiLSTM accuracy 97%, CNN+GRU 94%, CNN+LSTM 91%	Systematic representation-level comparison (raw audio vs. MFCC vs. spectral vs. Mel-spectral), unlike earlier works focusing only on spectrogram-based CNN transfer learning

## Data Availability

The datasets used and/or analyzed during the current study are available from the corresponding author on reasonable request.

## Abbreviations

The following abbreviations are used in this manuscript:

AUC	area under the curve
BiLSTM	bidirectional long short-term memory
CNN	convolutional neural network
DL	deep learning
DNN	deep neural network
EMD	empirical mode decomposition
FFT	fast fourier transform
GRU	gated recurrent unit
HC	healthy control
LSTM	long short-term memory
MFCC	Mel-frequency cepstral coefficients
ML	machine learning
PD	Parkinson’s disease
RPDE	recurrence period density entropy
SVM	support vector machine
